# A Gastrointestinal Stromal Tumor of the Stomach Demonstrating a Stepwise Progression from Low- to High-Grade Malignancy

**DOI:** 10.1155/2012/606832

**Published:** 2012-11-26

**Authors:** Takahiko Nakajima, Tomonori Ushijima, Atsushi Kihara, Kenichiro Murata, Toshiro Sugiyama, Koichi Tsuneyama, Johji Imura, Junichi Fukushima, Hajime Horiuchi

**Affiliations:** ^1^Department of Diagnostic Pathology, NTT Medical Center Tokyo, 5-9-22 Higashigotanda, Shinagawa-ku, Tokyo 141-8625, Japan; ^2^Department of Diagnostic Pathology, Graduate School of Medicine and Pharmaceutical Sciences, University of Toyama, 2630 Sugitani, Toyama 930-0194, Japan; ^3^Department of Gastroenterology and Hematology, Graduate School of Medicine and Pharmaceutical Sciences, University of Toyama, 2630 Sugitani, Toyama 930-0194, Japan

## Abstract

We report a case of a gastrointestinal stromal tumor (GIST) of the stomach that demonstrated a stepwise progression from low- to high-grade malignancy. The patient had been followed for a small gastric submucosal tumor that had turned malignant after 8 years of indolence, manifested by tarry stools. The tumor was enucleated, and gastric GIST was diagnosed. The most significant histological finding was that the tumor comprised two clearly demarcated areas, one with less aggressive characteristics and the other with highly aggressive characteristics. The patient exhibited multiple liver metastases 24 months after surgery. Imatinib mesylate was not administered throughout the clinical course because it was not available for clinical use at that time. The patient followed an unfavorable clinical course and died of liver dysfunction 55 months after surgery. Autopsy was performed. By comparing the immunohistochemical profiles of primary and metastatic tumors, it was established that only the tumor cells with highly aggressive characteristics had metastasized.

## 1. Introduction

The biology of gastrointestinal stromal tumors (GISTs) with regard to malignant characteristics is unclear. It is yet to be established whether malignant tumors are malignant from inception or become malignant after progression from preexisting, less aggressive tumors [1].

We experienced a case of gastric GIST; the tumor comprised two sharply demarcated areas, one with less aggressive characteristics and the other with highly aggressive characteristics. Thereafter, the case progressed to multiple metastases of the liver and peritoneum, and the patient died of liver dysfunction. Autopsy was performed. By comparing the primary and metastatic tumors, it was established that only the highly aggressive tumor cells had metastasized. This is an extremely rare case demonstrating histologically the stepwise progression of gastric GIST.

## 2. Case Presentation

A Japanese woman in her thirties was admitted with a chief complaint of tarry stools. Since the last 8 years, she had been followed for a small gastric submucosal tumor at a nearby hospital. She had undergone endoscopic examination at regular intervals and received no further treatment for the tumor; she remained symptomless during this period. Endoscopic examination at our hospital revealed a submucosal tumor with ulceration in the gastric antrum. Hemoglobin levels were 10.9 g/dL, and no metastatic lesions were found on computed tomography. The tumor was enucleated for treatment and diagnosis.

**Figure 1:**
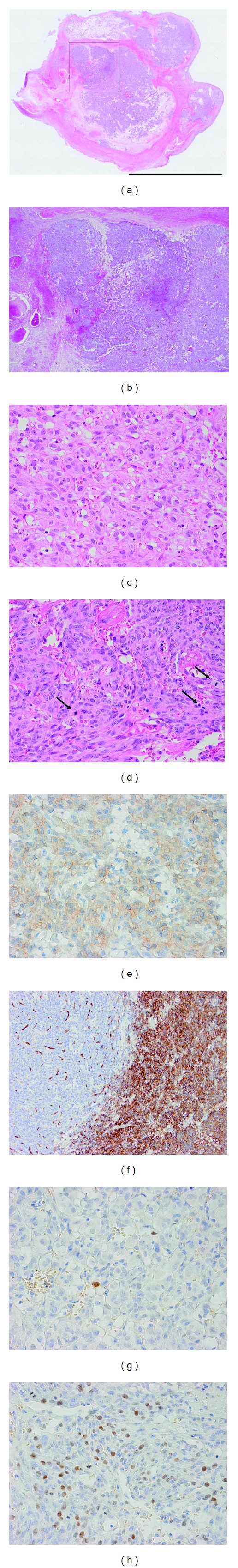
Light microscopic findings. (a) Whole-mount section of the tumor, with the ulcerated mucosal area to the left (hematoxylin and eosin, HE); bar, 1 cm. The area within the box is shown at a higher magnification in (b). (b) Highly aggressive tumor cells forming a demarcated area in the center, which is the only area where necrosis and hemorrhage were observed. The other areas comprised the less aggressive tumor cells (HE). (c) Higher magnification of the less aggressive tumor cells. The admixture of epithelioid and short spindle cells is shown (HE). (d) Higher magnification of the highly aggressive tumor cells. Note the higher cellular density and mitotic activity compared with (c). Mitotic activity is indicated by arrows (HE). (e) Tumor cells were immunopositive for KIT, irrespective of the area (KIT immunohistochemistry). (f) A clear demarcation line is shown by CD34 immunostaining. The positive and negative areas correspond to the less and highly aggressive tumor cells, respectively. (g) A low Ki-67 index is observed in the less aggressive areas. (h) A high Ki-67 index is observed in the highly aggressive area.

Grossly, the resected tumor was spherical with a maximal diameter of 1.9 cm. Microscopic examination revealed that the tumor comprised two distinct and clearly demarcated areas, one with less aggressive characteristics and the other with highly aggressive characteristics (Figures 1(a) and 1(b)). The highly aggressive tumor cells showed expansive growth (Figure 1(b)). An admixture of polygonal cells arranged in sheets and short spindle cells was observed, irrespective of the area involved. In the less aggressive areas, cellular density was moderate, with a mitotic count of 2 per 50 high-power fields (HPF) (Figure 1(c)). The highly aggressive area showed greater cellular density and a mitotic count of 31 per 50 HPF (Figure 1(d)). Necrosis and prominent hemorrhage were observed only in the highly aggressive area. Immunohistochemically, the tumor cells showed weak and diffuse positivity for KIT, which exhibited a combination of cytoplasmic and membranous patterns, and gastric GIST of mixed spindle-epithelioid cell types was diagnosed (Figure 1(e)). The less aggressive tumor cells were positive for CD34 and smooth muscle actin (SMA), with a Ki-67 index of 2% (Figures 1(f) and 1(g)), while the highly aggressive tumor cells were negative for CD34 and negative or weakly positive for SMA, with a Ki-67 index of 21% (Figures 1(f) and 1(h)).

The patient was recurrence-free for 24 months after surgery when multiple liver metastases were found. Transcatheter embolization, which is a method of treatment considered feasible prior to the clinical availability of imatinib mesylate, was performed but was ineffective. Thereafter, an unfavorable clinical course with peritoneal dissemination occurred, and the patient died 55 months after surgery. Autopsy was performed.

 At autopsy, large metastases in both liver lobes, multiple disseminated lesions of the peritoneum, necrosis, and hemorrhage were observed. Yellowish ascitic fluid (4.6 l) was also found. Microscopic examination of these tumors revealed a mixture of epithelioid and short spindle cells, with a mitotic count of 6 per 50 HPF. Immunohistochemically, the tumor cells were positive for KIT, negative for CD34, and negative or weakly positive for SMA, and the same result was observed for the highly aggressive tumor cells of the primary gastric lesion. The Ki-67 index was 5%. Consequently, the patient died of liver dysfunction.

## 3. Discussion

The tumorigenesis pathway of GIST is unclear. The malignant clone may originate from the less aggressive tumor, eventually rendering the whole tumor malignant as a result of clonal expansion, or the malignant tumor may be naturally malignant from the start of tumorigenesis. Our case supports the former theory of stepwise progression based on three significant findings. The first is that the gastric tumor comprised two areas of differing aggressive characteristics that were sharply demarcated, as clearly demonstrated by the differences in cellular density, mitotic rate, Ki-67 index, and necrosis, which are all powerful prognostic parameters for the risk assessment of GIST [2, 3]. The expansive growth of the highly aggressive tumor cells demonstrated clonal expansion. This case seems unlikely to be a collision tumor because the tumor was spherical, with the highly aggressive area located internally. The second is that only the highly aggressive tumor cells had metastasized. Finally, the tumor turned malignant after 8 years of indolence.

In a study comprising the largest ever number of gastric GIST cases with long-term follow-up, the correlation of immunohistochemical features and prognoses was analyzed. In that study, SMA-negative tumors showed a significantly higher frequency of progressive disease, and CD34-negative tumors showed a tendency to behave aggressively compared with CD34-positive tumors [4]. In our case, the preexisting tumor cells were positive for both SMA and CD34, while the tumor cells that metastasized were negative or weakly positive for SMA and negative for CD34. It is of great interest that the tumor cells changed their immunophenotypes with progression and that the observed changes correlated with the phases of indolent and aggressive clinical behavior. According to another study comprising the largest ever number of small intestinal GISTs, progression from a less aggressive tumor to a highly aggressive tumor was observed in 10 of 906 cases with histology of sharply demarcated areas [5]. However, in gastric GISTs, the frequency and occurrence of progression are unclear. We, therefore, believe that ours was an extremely rare case demonstrating the progression of gastric GIST that was positively proved by an immunohistochemical study comparing the primary and metastatic tumors.

If we assume that all malignant tumors arise from precursor tumors, the histological characteristics of progression would be observed more frequently. In practice, GISTs from surgical or autopsy specimens are generally large, and tumor progression manifested by two distinct areas is rarely encountered in pathological examinations. Progression may be complete before the tumor has become large and clinically detectable, with the entire tumor being dominated by the malignant clone. It is difficult to demonstrate this hypothesis because small tumors with aggressive characteristics, as in our case study, are rarely encountered. In the study comprising the largest ever number of gastric GISTs, 1552 tumors were grouped by size and mitotic activity, and only 8 tumors were grouped into the category of ≤2 cm with >5 mitoses per 50 HPF [4]. Some studies have reported on the molecular features that may be acquired in GISTs with tumor progression. Moreover, some studies have reported that the cytogenetic aberrations observed in malignant GISTs are in addition to those of less aggressive tumors [6–8]. Moreover, it is common for GISTs to acquire secondary resistance mutations in tyrosine kinase domains of the c-kit gene because they become refractory to imatinib therapy [9, 10]. These molecular findings support the theory of stepwise progression in GIST, but it remains unclear as to what stage with regard to size that tumors undergo the changes explaining progression to malignant behavior. The mechanisms of GIST progression, therefore, need to be further elucidated.

In conclusion, we experienced a case of malignant gastric GIST arising from a preexisting indolent tumor.
